# Assessment of *in vitro* potency of inactivated Newcastle disease oil-adjuvanted vaccines using hemagglutination test and blocking ELISA

**DOI:** 10.14202/vetworld.2018.1222-1228

**Published:** 2018-09-06

**Authors:** Saleh E. Aly, Hussein Ali Hussein, Abdel-Hakim M. Aly¹, Mansour H. Abdel-Baky, Ahmed A. El-Sanousi

**Affiliations:** 1 Central Laboratory for Evaluation of Veterinary Biologics, Abbasia 11381, Egypt; 2 Department of Virology, Faculty of Veterinary Medicine, Cairo University, Giza 12211, Egypt

**Keywords:** blocking ELISA, inactivated vaccines, *in vitro*, Newcastle disease virus, vaccine potency

## Abstract

**Aim:**

The present study was aimed to establish a protocol for the evaluation of the *in vitro* potency of commercial inactivated Newcastle disease virus (NDV) oil-adjuvanted vaccines using hemagglutination test (HA) and blocking ELISA (B-ELISA) based on polyclonal antibodies.

**Materials and Methods:**

Aqueous phases from a total of 47 batches of inactivated NDV vaccines manufactured by 20 different companies were extracted with isopropyl myristate. The viral antigen in each sample was detected and quantified by a standard HA test and a B-ELISA assay. To verify the efficiency of the antigen extraction method used in the batches which showed HA and to test the validity of using *in vitro* antigen quantification by HA and B-ELISA tests, a subset of 13 batches (selected from the total 47 batches) was inoculated in groups of 3-4-week-old specific pathogen-free chickens using the recommended vaccine dose. The immunogenicity of the selected vaccine batches was assessed by the NDV-hemagglutination inhibition antibody titers in individual serum samples collected 4 weeks after vaccination. Further, the efficacy of the vaccines and their protection rates were determined by a challenge test carried out for the vaccinated chickens with the Egyptian 2012 isolate of the virulent NDV genotype VII.

**Results:**

A strong correlation was observed between HA titers and B-ELISA mean titers in the tested 47 batches (R^2^=0.817). This indicated the possibility of using the latter *in vitro* assays for vaccine potency assessment. The recommended protective NDV antigen titer measured by B-ELISA was determined to be 28 ELISA units per dose. The comparison between the HA titers of the aqueous extracts of test vaccines and the corresponding results of *in vivo* potency assays (i.e., immunogenicity and efficacy), including antibody titers in the serum of vaccinated birds, indicated that the efficiency of the antigen extraction used may interfere with obtaining a strong correlation between the *in vitro* and *in vivo* results.

**Conclusion:**

HA or B-ELISA tests can be used as rapid and cost-effective alternatives to traditional *in vivo* potency tests for vaccine potency assessment by quantifying the NDV antigen present in aqueous phase extracts of the tested vaccines. The latter *in vitro* protocol, however, requires efficient extraction of the antigen to be able to obtain good correlation with the traditional *in vivo* potency tests.

## Introduction

Newcastle disease (ND) is a highly contagious viral disease that affects many avian species, especially chickens which exhibit one or more severe signs of septicemic gastroenteritis, bronchitis, and encephalitis. The disease is associated with high mortality [1]. ND is caused by a negative-sense single-stranded RNA virus of genus avian paramyxovirus (APMV) that belongs to the family Paramyxoviridae [2].

APMVs are classified into 15 different serotypes (APMV-1 to 15) according to their antigenic properties using a cross-hemagglutination inhibition (HI) test [3,4]. Serotyping of newly isolated strains can also be confirmed using phylogenetic analysis of their sequences. Known serotypes include APMV-10 [5], APMV-11 [6], APMV-12 [7], APMV-13 [8-10], and APMV-14 [11]. Recently, a novel serotype (APMV-15) was isolated from fecal droppings of wild birds at the UPO wetland in South Korea [12].

ND virus (NDV) is an endemic disease of chickens in Egypt, and the current policy of the disease control mainly depends on massive vaccination. Hundreds of batches of inactivated NDV oil-adjuvant monovalent and combined vaccines are submitted annually to the Central Laboratory for Evaluation of Veterinary Biologics (CLEVB) for reviewing their quality, wherein batch-by-batch release *in vivo* potency is granted.

Either an *in vivo* challenge test or an *in vitro* immunoassay [13] is used for routine potency assessment. A monoclonal antibody-based ELISA is usually the method of choice as a standard immunoassay for the *in vitro* potency assessment. To improve such assessment protocols, there is a need to implement the so-called “3Rs concept.” The latter comprises the concepts of (1) refinement of animal procedures, (2) reduction of animal numbers, and (3) replacement of animal models by utilizing serology instead of challenge and thus using antigen quantification instead of *in vivo* tests. The latter strategies have been widely recognized by the European veterinary authorities and thought to support the development and validation of alternative methods.

Currently, an *in vitro* potency assay is usually used to assess inactivated NDV oil-adjuvant vaccines (water in oil emulsion). A vaccine batch is approved or rejected based on quantification of the hemagglutinin-neuraminidase (HN) antigen content per dose in the aqueous phase of the vaccine after extraction with isopropyl myristate (IPM). The HN antigen is quantified using an in-house standard sandwich ELISA that relies on NDV HN monoclonal antibodies as a standard antigen assay. The results from the latter ELISA test correlates with *in vivo* potency assays [14-17]. However, such assay is of high cost and time-consuming. Hence, the development and establishment of new *in vitro* potency assays for inactivated NDV vaccines are essential for improving the process of monitoring and approval of vaccine batches before their release in the market. The latter target is of high priority for the CLEVB to save the time and cost of the *in vivo* potency testing procedure.

The present work was undertaken to illustrate the use of *in vitro* potency assays, namely hemagglutination (HA) test and blocking ELISA, in the evaluation of NDV vaccine efficacy, by quantification of the NDV antigen in aqueous phases of a total of 47 batches of commercial inactivated oil-adjuvanted monovalent vaccines. Comparison with *in vivo* potency assays was performed on a selected subset of those batches. In general, the use of liquid-phase blocking ELISA had been previously demonstrated in FMD use antigen [18].

## Materials and Methods

### Ethical approval

All institutional instructions and guidelines for the rearing and handling animals for research and experimental design have been followed.

### Experimental design

Test samples were collected from a total of 47 different batches of commercial inactivated NDV oil-adjuvanted vaccines. The selected samples represent the majority of locally manufactured and imported NDV inactivated vaccines in the Egyptian market. The aqueous phases of the samples (containing the viral antigen) were extracted with IPM. The NDV antigen was detected and quantified by HA test and B-ELISA. A selected subset of 13 batches (from the total of 47 batches) was tested for their immunogenicity and efficacy using an *in vivo* potency assay in specific pathogen-free (SPF) chickens. The latter 13 batches included samples/batches that were found to be invalid batches using the *in vitro* potency assays (i.e. included those vaccine batches that gave low antigen readings when quantified by HA and B-ELISA tests).

### Vaccines

Samples from a total of 47 batches of commercial inactivated NDV oil-adjuvanted monovalent vaccines manufactured by 20 different companies that were submitted to CLEVB, Cairo, Egypt, between 2015 and 2017, were selected and used for this study. The samples were used for the assessment and evaluation of the proposed *in vitro* potency protocol in which HA test and B-ELISA were used as alternatives for the traditionally used methods for NDV vaccine potency assessment, including monoclonal sandwich ELISA and *in vivo* challenge tests. The results were used to compare the new protocol with the traditional *in vivo* potency testing methods.

### Viruses

A velogenic NDV, isolate D7.RLQP.CH.EG.12, GeneBank accession no: KM288609 supplied by Reference Laboratory for quality control of poultry production (RLQP), El-Giza, Egypt, was propagated and titrated in 9-11-day-old SPF-ECEs. The strain was checked for its identity, purity, and pathogenicity by the CLEVB. This strain was used as the challenge NDV for testing the efficacy of the vaccine batches. A lentogenic NDV (LaSota strain) obtained from a validated commercial live vaccine, and propagated in 9-10-day-old SPF-ECEs, was used for the preparation of the HA antigen.

### Extraction of NDV antigen from inactivated oil-adjuvanted vaccines

A 2 mL volume of the test vaccine was taken using a sterile syringe from a well-shaken vaccine container and mixed with 8 mL IPM-98% (lot #114181205, LOBA chemic, India) in sterile polypropylene centrifuge tube and shaken on a vortex of maximum speed 2500 rpm for 1 min. The mixture was then centrifuged at not <1000× *g* for 10 min at 8°C. Carefully, the lower aqueous phase was collected and immediately used for the HA test and blocking ELISA to quantify the extracted viral antigen.

### HA and HI tests

Standard HA and HI tests were performed in U-shaped bottom 96-well microtiter plate to quantify and identify HA activity of each vaccine extract. Briefly, for the HA test, 2-fold serial dilutions of the aqueous extracts of the test vaccines and the positive control LaSota antigen were made using sterile phosphate buffer saline (PBS) as a diluent, pH 7.2, starting with 50 μL of the extract or positive antigen and 50 μL of PBS, and up to dilution eleven. Dilutions were made in duplicates of each (2 wells/dilution). A fixed amount (50 μL) of approximately 0.5% washed chicken erythrocytes (red blood cells [RBCs]) was added to each test well as well as to extra wells containing PBS only (RBCs controls). The plates were shaken gently, covered and incubated at room temperature for approximately 30 minutes until agglutination occurred in the positive antigen control wells, and until precipitation occurred in RBCs control wells. HA was read by tilting the plate and observing the presence or absence of tear-shaped streaming of the RBCs. HA titer of each test extract and positive control antigen was calculated as the mean of the reciprocals of the highest dilutions given complete HA (no streaming).

The HI test was performed as follows: 2-fold serial dilutions were made from the positive antigen control and from each of the test extracts which gave positive results for HA with titers not <32. The serial dilutions were made in two rows across wells between 1 and 12 of the microtiter plate (50 μL/well) using PBS, pH 7.2. In one row, an amount of 50 μL of the diluted standard anti-NDV chicken serum, that is, sufficient to inhibit NDV of >8 HA units, was dispensed into each well. The other rows were compensated by the addition of 50 μL PBS/well. After gentle shaking, the plates were covered and incubated at room temperature for approximately 45 min. Thereafter, an amount of 50 μL of 0.5% washed chicken RBCs was dispensed to each of the working wells. Again, after gentle shaking, the plates were covered and incubated at room temperature until RBCs control wells formed distinct button shape. The plates were subsequently examined for the presence or absence of tear-shaped streaming of RBCs.

The extract HA activity was considered identical to NDV-HA activity if the test extract-antiserum and LaSota antigen-antiserum mixtures showed HI titer not <32 and the non-neutralized extract showed its predictive HA titer.

### Blocking (B)-ELISA

B-ELISA was conducted to quantify NDV antigen titers in aqueous extracts of the test vaccines as well as positive and negative controls. The NDV LaSota antigen was used as a positive antigen control while aqueous extracts of two commercial inactivated avian influenza viruses, subtype H5N1 oil-adjuvanted vaccines were used as negative antigen controls. The test was carried out using the commercial Symbiotic Proflok Plus NDV antibody detection indirect ELISA test kit, Batch no.14016648, and standard anti-NDV chicken serum. The test was carried out as follows:

The test extracts, as well as the positive and negative antigen controls, were diluted in 2-fold serial dilutions between 1/2 and 1/256 in U-shaped bottomed 96-well polypropylene microtiter plate (transferring plate) starting with a 100 μL of the extract or control antigen and a 100 μL of the kit diluting buffer. The kit positive control chicken serum or standard anti-NDV chicken serum was diluted as recommended by the kit manufacturer or as pretested. The latter was added to each of the diluted extract and control antigen (50 μL/well), and then the plates were shaken gently, covered and kept overnight at 4°C. The diluted extract-antiserum mixtures and the negative and positive antigen-antiserum mixtures were then transferred from the transferring plate into the NDV antigen precoated ELISA kit plate (two wells/mixture, each 50 μL/well, i.e., samples were tested in duplicates). Each of the diluted positive and negative controls was also dispensed in two wells and then plates were covered and incubated at 37°C for 60 min. The ELISA test was completed by adding the anti-species labeled antibodies, following the kit instructions. The antigen titer is expressed as the reciprocal of the highest dilution given that the mean optical density (OD) reading value is not more than 50% of the mean OD reading of the positive control serum. The test plate was not valid unless both of the negative and positive control sera recorded OD values that are within the lower and upper limits of the test kit, i.e. the positive antigen-antiserum mixtures recorded its predictive titer ±1 log_2_, and the negative antigen-antiserum mixture gave negative readings. The vaccine NDV-HA antigen and ELISA antigen units per dose were calculated as follows:


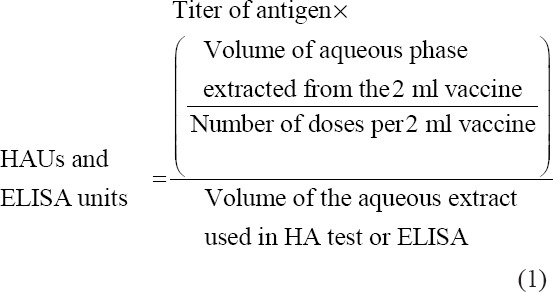


### Immunogenicity and efficacy tests

Based on results of *in vitro* potency assays of the tested 47 batches of inactivated NDV oil-adjuvanted vaccines, 13 batches of the 47 were selected and inoculated in SPF chickens of 3-4 weeks old using the recommended vaccine dose and recommended route of injection (15 birds/group). An additional group of chickens from the same source and age was kept as a negative control group. Hence, a total of 210 SPF chickens of 3-4 weeks were used for the immunogenicity and efficacy tests of the 13 selected batches. All chicken groups were maintained in battery cages in separate units.

28 days after vaccination, individual blood samples were collected from the vaccinated and unvaccinated chickens, and NDV-HI antibody titers in the obtained serum samples were measured by HI test. The vaccine was considered immunogenic (i.e., valid) if the geometric mean of the HI antibody titer is not <6 log_2_ in the sera of the vaccinated chickens. The sera of negative control chickens did not record HI titers of more than 2 log_2_.

Six chicken groups from the 13 vaccinated groups, as well as the unvaccinated chickens, were challenged by intramuscular injection of the virulent NDV, isolate B7. RLQP.Ch.Eg12, (6 log_10_ ELD_50_/bird). Challenged chickens were observed daily for 14 days after inoculation of the challenge virus. Percent of protection was calculated for each group of the vaccinated and unvaccinated challenged chickens. The vaccine was considered efficacious (valid) if not <90% of the vaccinated challenged chickens survived without showing clinical evidence of NDV, and at least 90% of unvaccinated challenged chickens died during the first 6 days of the entire observation period [18].

### HI test (HI) for *in vivo* tests

HI test was carried out to measure NDV-HI antibody titers in sera of vaccinated and unvaccinated chickens using 2-fold serial dilutions of the test and control serum samples between 1/2 and 1/2048 in duplicates against four HAUs of NDV LaSota antigen [1]. Geometric mean HI antibody titer of each chicken group was calculated by the method used in reference [19].

## Results

Table-1 [20] demonstrates the corrected titers (using Eq. 1) of NDV antigen/dose from aqueous phases extracted from the samples of 27 batches, extracted with IPM, and the antigen was quantified by HA and B-ELISA. The latter batches are from 12 different commercial inactivated oil-adjuvant vaccines. HAUs per dose showed a very wide range, as low as 2 to >4128 HAU/dose. In previous studies, the recommended HAUs cutoff value of the potent NDV inactivated vaccines was reported to be 500 HAUs/dose [20]. Within the tested batches, 12 of 27 batches (44 %) were above the cutoff, with a minimum of 716 HAUs and their corresponding geometric mean titer (GMT)-ELISA units per dose were found to be >30. On the other hand, 15 of 27 batches (56 %) showed titers that are below the cutoff and had ranged between 120 and <2 HAUs. Hence, the NDV antigen was extracted successfully from at least 12 of the 27 test batches (44 %) as measured with HA test and B-ELISA.

**Table-1:** Frequency of batches contained antigens within various ranges.

Number of batches	HA units/dose	B-ELISA units/dose
1	≥4128	≥356
3	3840	178
2	3584	82
2	2445	58
2	1920	38
1	1664	30
1	716	30
2	120	25
2	20	12
1	14	10
10	2	6

Quantities of NDV antigen per dose in aqueous phases of a total of 27 batches of commercial inactivated oil-adjuvanted vaccines from 12 different manufacturers as extracted with IPM and tested by HA and B-ELISA. The recommended cutoff value of NDV antigen in a potent inactivated oil-adjuvanted vaccine is 500 HAUs [20]. HA=Hemagglutination, NDV=Newcastle disease virus, IPM=Isopropyl myristate

To ensure the accuracy of NDV antigen extraction with IPM, samples from 10 different batches, the extraction and the antigen detection using HA was repeated for 3 times. Only one 2-fold increase or decrease of the HA titers was observed in only one sample, demonstrating that the error in the tests is estimated to be within the range of 0-10% (un-tabulated results).

**Figure-1:**
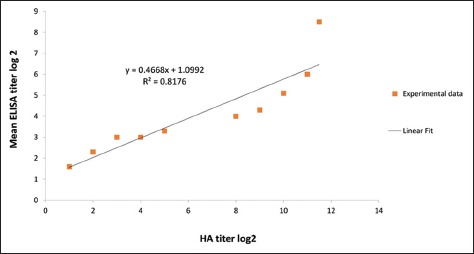
Correlation between hemagglutination (HA) and B-ELISA titers. The figure shows a plot of HA versus B-ELISA titers of Newcastle disease virus antigen. The titers are measured from aqueous extracts of a total of 47 batches from 20 different commercial inactivated oil-adjuvanted vaccines. The linear relationship indicates a correlation between HA and B-ELISA titers.

In correlation with HAUs records of the test extracts, NDV antigen-ELISA units per dose in 12 of the 27 test extracts ranged between 30 and >356 ELISA units. The latter was above the value of the protective HAUs cutoff. Hence, the protective cutoff in HA (which is 500 HAUs) corresponds to ≤30 ELISA units (~28 ELISA units) in B-ELISA. On the other hand, 15 out of the 27 test extracts gave ELISA titers that ranged between 6 and 25 ELISA units. The latter range is below the cutoff value. The comparison between the obtained HA titers and the arithmetic mean of B-ELISA titer, in all the 47 samples (Table-2), showed linear correlation with a residual value (R^2^) of 0.817 (Figure-1).

**Table-2:** Correlation between HA and B-ELISA titers of NDV antigens.

Number of batches	HA (titer in log_2_/50 μl)	B-ELISA (arithmetic mean titer in log_2_/50 μl)
2	11	≥8.5
6	10	6.0
6	9	5.1
3	8	4.3
2	5	4.0
6	4	3.3
2	3	3.0
2	2	3.0
4	2	2.3
14	<2	1.6

The table shows the correlation between HA and B-ELISA titers of NDV antigens. The reported titers were obtained by quantifying antigens from aqueous extracts of a total of 47 batches from 20 different commercial inactivated oil-adjuvanted vaccines. HA=Hemagglutination, NDV=Newcastle disease virus

Based on the obtained results of *in vitro* potency assays, 13 from the total of 47 test vaccines were selected for *in vivo* potency assays (immunogenicity and efficacy) by inoculation in different groups of SPF chickens (see materials and methods). The results showed that five batches contained HA antigen titer <1 log_2_, three batches contained 1 log_2_, three batches had 5 log_2_, and two batches recorded 10 log_2_ and 11 log_2_/50 μL. These vaccines batches induced the production of the corresponding GMT of NDV-HI antibodies of (4.9, 32, 69, 121, and 446), (79, 97, and 362), (79, 91, and 208), and (194 and 181) in sera of the vaccinated chickens, respectively (Table-3).

**Table-3:** *In vivo* potency assays of commercial inactivated NDV oil-adjuvanted vaccines.

HA titer (log_2_) exhibited by 50 μls of the vaccine extract	GMT of HI antibodies in sera of the vaccinated chickens	% protection
1-2	4.9	NT
1-2	32	90
1-2	69	NT
1-2	121	100
1-2	446	100
2	79	NT
2	97	100
2	362	NT
5	79	NT
5	91	NT
5	208	100
10	194	100
11	181	NT

The table shows the results of *in vivo* potency assays of 13 different batches of commercial inactivated NDV oil-adjuvanted vaccines as determined by the GMT of HI antibodies in sera of vaccinated chickens. The corresponding % protection against challenge with virulent NDV is also tabulated. “NT” indicates that batches were not “Not tested”. It should be noted that unvaccinated challenged chickens group exhibited 90% mortality. HA=Hemagglutination, NDV=Newcastle disease virus, HI=Hemagglutination inhibition, GMT=Geometric mean titer

To confirm the vaccine potency obtained from the measured titers, six groups of previously vaccinated chickens (of the total of 13 selected vaccine batches) were challenged with the virulent Egyptian 2012 isolate of genotype VII. The six batches were able to protect the vaccinated chickens against the challenge virus morbidity, and mortality at rates ranged between 90% and 100% (Table-3). It can be observed from the latter table that no direct correlation exists between HA titers of extracted antigens and GMTs of HI antibodies from the sera of vaccinated chickens. Similarly, there was no direct correlation between the HA titers of extracted antigens and the protection rates of the vaccinated chickens. This lack of correlation is possibly due to variations in the degree of antigen extraction between commercial vaccines (see discussion for details).

## Discussion

The development and establishment of *in vitro* potency assays for batches release of inactivated NDV vaccine are of high priority for Central Laboratory for Evaluation of Veterinary Biologics (CLEVB) to save time and reduce the cost of *in vivo* potency testing. Strict batch-by-batch release potency assays of NDV vaccines are undertaken by the CLEVB. Infectious dose 50 assays for each batch of the live vaccines and *in vivo* potency assays for batch release of inactivated vaccines based on vaccination of 3-4-week-old SPF chickens with a full dose are routinely performed to assess the potency of commercial vaccines. In the *in vivo* potency tests, a GMT of ≥6 log_2_ of HI antibodies in serum samples collected 3-4 weeks after vaccination [1] is required for approval. Otherwise, if the GMT of HI antibodies was <6 log_2_ and ≥4 log_2_, a full dose vaccination challenge model is carried out, and a requirement of at least 90% protection is needed for approval and batch release [18].

Several studies were previously conducted to develop *in vitro* potency assays attempting to replace *in vivo* tests, by quantification of the NDV antigen content after the treatment of the vaccine with IPM to separate the aqueous phase that contains virus antigen [16,17]. Using modified B-ELISA methods have proven to be very useful to reduce the time and cost compare to conventionally used methods and compared to methods [19,21] that depend on monoclonal antibodies [14,22] in NDV vaccines monitoring and batch release. Other *in vitro* methods such as single radial immunodiffusion had been used for the same purpose. The latter method is one of the most beneficial and rapid methods for measurement potency of influenza vaccines [23-25], but with the downside that it needs extensive optimization and standardization. Moreover, mass spectrometry [26] and size-exclusive high-performance liquid chromatography [27] were also used as alternative methods for vaccines potency evaluation, by antigen purification and quantification. However, such methods are of high cost and time-consuming.

The present study was performed to establish a protocol that can reduce or substitute *in vivo* potency testing. Samples of 47 batches of inactivated NDV oil-adjuvanted vaccines manufactured by different companies were treated with IPM, leading to the extraction of the antigen which was subsequently quantified using HA/HI test and B-ELISA. Variable NDV-HA titers that ranged between 11 log_2_ and <2 log_2_/50 μL had been observed in approximately 70% of the 47 extracts. A titer of 500 HA units per dose was taken as a cutoff quantity of inactivated NDV-HN antigen to grant the commercial vaccine as a potent vaccine [20]. In the tested samples, 44% of the corrected 27 antigen titers were found to contain more than 500 HAUs per dose. The latter HA titers per dose ranged between >4128 and 716 HAUs.

On the other hand, B-ELISA has succeeded in detecting NDV antigen in 100% of the sample extracts which showed a wide range of mean titers (>8.5 log_2_-1.6 log_2_/50 μL). A linear correlation between the average mean ELISA titers ranging from 5 log_2_ to 2.3 log_2_ and the average HA titers ranging from 10 log_2_ to 2 log_2_ were observed. In fact, this correlation was, as shown in Figure-1, around the protective HA titer cutoff. The protective NDV antigen ELISA cutoff GMT value of the present study was calculated and found to be 28 units per dose. Based on the differential cutoff value of HAUs and its correlated B-ELISA units of NDV antigen in aqueous phases extracted by IPM, and obtained from different test vaccines, more than 60% of these vaccines were apparently considered impotent and this matter was questionable. Hence, *in vivo* potency tests were done to verify the potency of 11 different selected batches which scored HA titers between <2 log_2_ and 5 log_2_ plus two batches which showed HA titers of 10 log_2_ and 11 log_2_/50 μL as positive control vaccines using the vaccination antibody response assay (immunogenicity) for the 13 batches. Further, vaccination challenge (efficacy) for 6 of the 13 batches was performed.

*In vivo* potency assays results revealed that only one batch of the 13 batches was impotent. There was no correlation between the obtained HA titers of the extracts of 10 batches and it is *in vivo* potency assays. Several reasons may have caused this lack of correlation. Since these vaccines are from different manufacturers, they may contain different oil adjuvants. The exact nature and type of the oil emulsion used by different manufacturers of the test vaccines were not known because the test samples had been received as coded samples. It should be noted that the nature and type of the oil emulsion in many of the test vaccines might have interfered with the efficiency of the extraction process with IPM, and the extracted viral glycoproteins may still be partially coated/masked by the adjuvant or the extraction substance. Therefore, the present study did not guide us to specify if the extraction with IPM is efficient for certain commercial vaccines and not for others. However, the study had shown that in the case of efficient extraction, the antigen quantification by HA and B-ELSA could substitute *in vivo* potency testing. Further, the study has also shown that these *in vitro* tests, if done before any *vivo* investigations, can help to illuminate up to 44% of the routine *in vivo* tests. If the GMTs recorded a value below the recommended cutoff value, only then, an *in vivo* potency assay must be performed. Moreover, the study has illustrated that the time and cost required for the *in vitro* tests (for antigen quantification) can be reduced using B-ELISA based on polyclonal instead of the conventional monoclonal antibody-based ELISA.

## Conclusion

The present study demonstrated that quantification of the extracted NDV antigen by HA test or B-ELISA (*in vitro* potency assays) after the treatment of the commercial inactivated oil-adjuvanted vaccines with IPM could be used as an alternative method or at least as a supplemental method for traditional *in vivo* potency tests used for batch release and vaccine potency assessment. These methods make a protocol that has been shown to be simple and effective and was able to reduce the time and cost of the routine *in vivo* assays. Moreover, the protocol is a suitable choice if a rapid check is needed as in the case of emergency or in the case of monitoring the keeping and storage quality (shelf life) of such vaccines. B-ELISA has an extra advantage over HA and HI in that it can be useful for *in vitro* potency assays of inactivated combined oil-adjuvanted vaccines of NDV with other viruses. *In vitro* potency assays for batch release of NDV inactivated oil-adjuvanted vaccines may be based on the quantification of NDV antigen in extracted aqueous phases, from samples obtained from at least three containers of the test vaccine, using HA test or B-ELISA. For a vaccine to be considered potent, the geometric mean antigen titer is required to be not <500 HAUs/dose or its equivalent ELISA units. In the latter case, no further *in vivo* tests are required. If the antigen titer was, however, less than the required titer, *in vivo* potency tests would be required. The reliability of *in vitro* tests will likely to increase with the development of more efficient antigen extraction methods, which by current methods may vary between one commercial vaccine and another.

## Authors’ Contributions

This work was a part of the Ph.D. thesis of SEA supervised by AAE, HAH, AMA, and MHA. SEA: Conducted the laboratory animal experimental work, drafted, and revised the manuscript. MHA and AMA: Contributed to the design of the experimental work and followed up the practical part of the research. AAE and HAH: Designed the experiments, supervised the work, drafted, and revised the manuscript. All authors have read and approved the final manuscript.
